# Autonomous Lunar Rover Localization while Fully Scanning a Bounded Obstacle-Rich Workspace

**DOI:** 10.3390/s24196400

**Published:** 2024-10-02

**Authors:** Jonghoek Kim

**Affiliations:** System Engineering Department, Sejong University, Seoul 05006, Republic of Korea; jonghoek@gmail.com

**Keywords:** rover localization, Lidar, location estimate fix, dark outer space rover, base station, space robot, coverage path plan, scanning path plan

## Abstract

This article addresses the scanning path plan strategy of a rover team composed of three rovers, such that the team explores unknown dark outer space environments. This research considers a dark outer space, where a rover needs to turn on its light and camera simultaneously to measure a limited space in front of the rover. The rover team is deployed from a symmetric base station, and the rover team’s mission is to scan a bounded obstacle-rich workspace, such that there exists no remaining detection hole. In the team, only one rover, the hauler, can locate itself utilizing stereo cameras and Inertial Measurement Unit (IMU). Every other rover follows the hauler, while not locating itself. Since Global Navigation Satellite System (GNSS) is not available in outer space, the localization error of the hauler increases as time goes on. For rover’s location estimate fix, one occasionally makes the rover home to the base station, whose shape and global position are known in advance. Once a rover is near the station, it uses its Lidar to measure the relative position of the base station. In this way, the rover fixes its localization error whenever it homes to the base station. In this research, one makes the rover team fully scan a bounded obstacle-rich workspace without detection holes, such that a rover’s localization error is bounded by letting the rover home to the base station occasionally. To the best of our knowledge, this article is novel in addressing the scanning path plan strategy, so that a rover team fully scans a bounded obstacle-rich workspace without detection holes, while fixing the accumulated localization error occasionally. The efficacy of the proposed scanning and localization strategy is demonstrated utilizing MATLAB-based simulations.

## 1. Introduction

This article addresses the scanning path plan strategy of a rover team composed of three rovers, such that the team explores unknown dark outer space environments. Here, scanning path plan is to determine a rover’s path passing over every point of a given bounded obstacle-rich workspace, such that there is no detection hole. We consider the situation where an obstacle environment in outer space is not known in advance.

This research considers a dark outer space, where the Sun light and the Global Navigation Satellite System (GNSS) are not available. Thus, a rover needs to turn on its light and camera simultaneously to measure a limited space in front of the rover. The rover team is deployed from a symmetric base station, and the team’s mission is to scan a bounded obstacle-rich workspace, such that there is no remaining detection hole. This scenario was inspired by the NASA Space Research Challenge Phase 2 [1,2].

The purpose of the NASA Space Research Challenge Phase 2 was for the competitors’ virtual multi-robot teams to complete tasks for a lunar In-Situ Resource Utilization (ISRU) mission. This challenge allowed a virtual multi-robot team to operate autonomously for achieving these tasks. On 15 January 2021, NASA selected the Purdue-Hongik Team (the author is a member of the team) as a qualifying team to advance to the final virtual competition round [3]. In the competition, the rover team’s mission was to make a virtual robot team detect, excavate, and gather underground resources to the base station. For maximizing the efficiency of resource gathering, it is desirable to scan a bounded obstacle-rich workspace without detection holes. This paper was written inspired by the competition.

**Figure 1:**
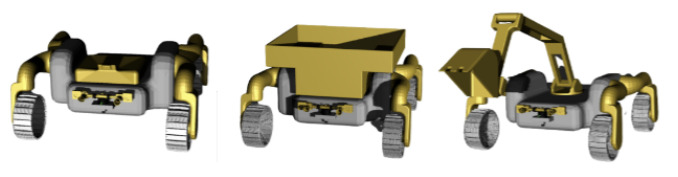
The NASA Space Research Challenge [1] considered a team of three rovers: the scouter, the hauler, and the excavator. From the left to the right in this figure, one depicts the scouter, the hauler, and the excavator in this order.

The NASA Space Research Challenge [1,2] considered a team of three rovers: the scouter, the hauler, and the excavator. See Figure 1 for an illustration of these three rovers. From the left to the right in this figure, one depicts the scouter, the hauler, and the excavator in this order.

In the NASA Space Research Challenge, the scouter, the hauler, and the excavator are used for the excavation and the collection of resources from the Moon [1]. Only the scouter has sensors for detecting resources buried in the ground using its local sensors. Once the scouter detects resources, the detected resources are excavated by the excavator, and the resources are dropped into the bin of the hauler. See Section 4.4 for controls of the excavator.

In order to enable cooperation among three rovers, communication link among three rovers needs to be established at all times. Thus, all three rovers need to move as a team. In the team, we assume that only the hauler can locate itself utilizing stereo cameras and Inertial Measurement Unit (IMU). Every other rover follows the hauler, while not locating itself. In this way, both the scouter and the excavator do not have to run localization process which can be computationally heavy. See Section 4.5 for controls of hauler following maneuvers.

**Figure 2:**
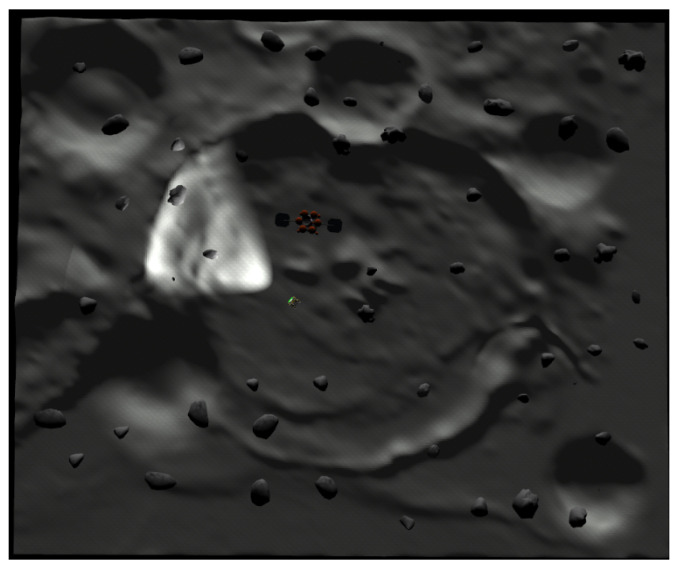
The bounded obstacle-rich workspace of the NASA Space Research Challenge. Rocks are presented as obstacles of MATLAB-based simulations (see Section 5). The rover turns on its light in dark environments.

The bounded obstacle-rich workspace of the NASA Space Research Challenge is presented in Figure 2. Rocks are presented as obstacles of MATLAB-based simulations (see Section 5). In Figure 2, the rover turns on its light in dark environments.

In order to scan a bounded obstacle-rich workspace, one requires localization of a rover. The lack of distinct features on lunar surface makes the localization problem further challenging and requires solutions that are catered specifically to the lunar rovers.

Moreover, the NASA Space Research Challenge provides a dark workspace, such that a rover needs to turn on its light and camera simultaneously to measure the space in front of the rover.

While the rover with cameras maneuvers, it can localize itself utilizing various localization methods in the literature. Considering many environmental factors (surface flatness or soil softness), odometry system of a rover can be used for the rover’s localization [4,5]. See that lunar surface in Figure 2 is not even, and localization error of a rover can increase considerably while a rover moves on hills or mountains. Therefore, localization error of odometry system must integrate as time goes on [4].

Visual-inertial simultaneous localization and mapping (VI-SLAM) [6,7] or monocular SLAM [8,9,10] can be utilized for rover localization in real-time. The authors of [11] addressed a real-time algorithm generating the 3D path of a monocular camera, moving through a previously unknown scene. VI-SLAM integrates camera and IMU for a rover’s localization [6,7]. But, the position accuracy, computational load, and memory burden of this VI-SLAM remain open challenges [7]. Under VI-SLAM, it is inevitable that localization error integrates as time elapses, as long as a loop closure is not observed.

VI-SLAM requires that the rover can detect reliable environmental features. Features widely utilized in VI-SLAM are ORB, SIFT, and SURF [6]. However, the lack of distinct features on lunar surface makes the localization problem further challenging and requires solutions that are catered specifically to the lunar rovers.

As far as we know, only [12] addressed the exploration of three rovers (the scouter, the hauler, and the excavator) in dark outer space environments. However, [12] didn’t present a strategy for covering the bounded obstacle-rich workspace, as in our research.

Considering the exploration of a dark outer space, [12] applied VI-SLAM, which is based on stereo cameras and IMU. By applying any SLAM approaches in dark outer space environments, it was inevitable that localization error integrates as time elapses [12]. This is due to the fact that distinct and stable features do not exist on lunar surface. Moreover, the rover can only measure a limited space in front of the rover by turning on its light and camera simultaneously. Considering outer space environments where the terrain is not flat (see Figure 2), tire slippage can further increase the localization error of SLAM algorithms. For fixing the integrated localization error of a rover, [12] occasionally makes the rover home to a symmetric base station, whose shape and global position are known in advance.

**Figure 3:**
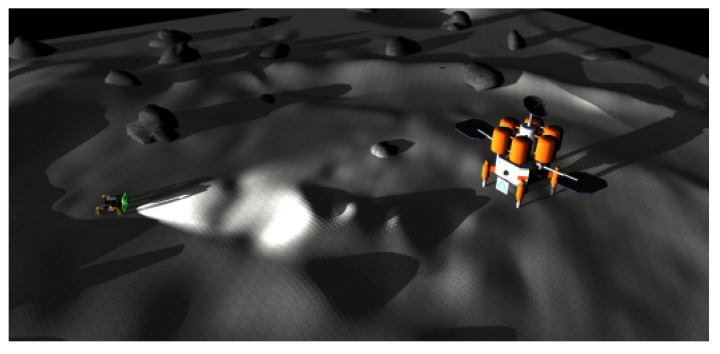
This figure plots a symmetric base station used in the NASA Space Research Challenge [1]. The rover turns on its light in dark environments. To the right of the rover in this figure, there is the symmetric base station. It is assumed that the station’s global position and shape are known in advance.

For example, Figure 3 plots a symmetric base station used in the NASA Space Research Challenge [1]. In Figure 3, the rover turns on its light in dark environments. In Figure 3, there is the symmetric base station to the right of the rover. It is assumed that the station’s global position and shape are known in advance.

Once the rover is near the base station, it turns on its Lidar to measure the relative position of the base station. The Lidar provides the relative position of the base station with respect to the rover. This relative position information provides the global localization for the rover. Suppose that the base station is at the origin, and that the relative position information of the base station with respect to the rover is $R_{r}$. In this case, the global position of the rover is estimated as $-R_{r}$. In this way, the rover fixes its localization error whenever it homes to the base station. This homing strategy diminishes the integrated location error to zero.

This homing strategy is used in our research, so that localization error can be fixed occasionally. In our research, one makes the rover team scan a bounded obstacle-rich workspace without detection holes, such that a rover’s location error is bounded above. For diminishing the integrated location error to zero, one makes a rover home to the base station occasionally. We acknowledge that returning to the base station can slow the exploration progress.

In our paper, a rover senses a nearby obstacle based on stereo cameras or Lidar, as presented in [12,13]. In Figure 2, rocks are presented as obstacles of MATLAB-based simulations (see Section 5). A rover can use Lidar or stereo cameras to determine if it senses a large obstacle in front of it [12,13]. In our research, one makes the rover team fully scan a bounded obstacle-rich workspace without detection holes, such that a rover’s location error is bounded and that a rover avoids collision with other obstacles or other rovers.

To the best of our knowledge, our article is novel in addressing the scanning path plan, so that the rover team (the scouter, the hauler, and the excavator) fully scans a bounded obstacle-rich workspace while fixing the location error occasionally. In the rover team, only the hauler needs to locate itself utilizing stereo cameras and IMU. Moreover, only the scouter has the ability to scan the space searching for buried resources. The efficacy of the proposed scanning and localization strategy is demonstrated utilizing MATLAB-based simulations.

The organization of this article is as follows. Section 2 presents the literature review related to this research. Section 3 presents the assumptions and definitions in this research. Section 4 addresses the scanning path plan strategy introduced in this research. Section 5 addresses MATLAB-based simulation results to demonstrate the performance of the proposed strategy. Section 6 provides conclusions.

## 2. Literature Review

As far as we know, only [12] addressed the exploration of three rovers (the scouter, the hauler, and the excavator) in dark outer space environments. In order to make a rover detect every resource buried in the ground, we need to make the rover scan the bounded obstacle-rich workspace without detection holes. However, [12] didn’t address a strategy for covering the complete outer space workspace using a rover team. In other words, [12] didn’t address a strategy for planning the path of rover team, so that the bounded obstacle-rich workspace is scanned without detection holes. See Figure 10 in [12]. In the team, [12] assumed that every rover needs to locate itself utilizing stereo cameras and IMU. However, our research assumes that only the hauler locates itself utilizing stereo cameras and IMU.

There are many papers on scanning path plans to cover the entire workspace [14,15,16,17,18]. The authors of [14] addressed a literature review on the scanning path plan. The reference [15] addressed underwater sonar surveys using multiple sonar-equipped autonomous surface vehicles, such that vivid sonar images can be derived while covering the bounded obstacle-rich workspace completely. The authors of [16] addressed an algorithm for building a scanning plan over a holed rectilinear polygonal region. Reference [17] introduced a scanning path plan for scanning a spill according to aerial images from remote sensing. Reference [18] addressed the boustrophedon cellular decomposition, such that every boustrophedon cell is scanned with simple backwards and forwards maneuvering. References [19,20,21,22] applied lawnmower parallel path planning for scanning the given workspace.

However, to the best of our knowledge, no paper in the literature considered localization of a rover, while the rover fully scans the complete open space. In other words, other papers on scanning path plans didn’t consider the fact that a rover’s localization error will increase as time goes on, as long as the location estimate fix is not performed.

Several papers [23,24,25] showed that random maneuvers can be used to find resources in the workspace. For random search, Levy walks, brownian motion, or ballistic (straight line) search with random bounce can be utilized. Suppose that rovers move as a group and that they move randomly in the workspace, while avoiding collision with obstacles. As we use this random search strategy, complete coverage of a bounded obstacle-rich workspace can’t be assured. Moreover, the rovers’ location error integrates as time goes on. Using MATLAB-based simulations, we show that the proposed scanning strategy outperforms this random maneuver strategy.

To the best of our knowledge, our article is novel in addressing the scanning path plan, so that the rover team (the scouter, the hauler, and the excavator) fully scans a given workspace while fixing the location error occasionally. Only the hauler can locate itself utilizing stereo cameras and IMU. Moreover, only the scouter has the ability to scan the space searching for buried resources.

## 3. Assumptions and Definitions

A graph *G* is a set G=(V(G),E(G)), where V(G) is the vertex set and E(G) is a set of edges. Each edge, say e∈E(G) has its weight as $w\left(e\right):e\to Z^{+}$.

Let *R* stand for the hauler positioned at $R\in R^{2}$. Let $R^{s}$ stand for the scouter positioned at $R^{s}\in R^{2}$. Let $R^{e}$ stand for the excavator positioned at $R^{e}\in R^{2}$.

Let $r_{s}$ stand for the sensing range limit of a rover. For planetary rovers, obstacles include rocks, loose soil, or regolith that causes the wheels to slip and potentially get stuck [26]. A rover senses a nearby obstacle based on stereo cameras or Lidar in dark space, as presented in [12,13]. A rover can detect an obstacle or another rover, that is within $r_{s}$ distance from the rover. By doing experiments using stereo cameras or Lidar in dark space, $r_{s}$ can be determined. We acknowledge that the light intensity of a rover can make effects on $r_{s}$. As the light intensity increases, stereo cameras can detect an obstacle which is far from the rover. Thus, as the light intensity increases, $r_{s}$ increases as well. A *colliding occurs* in the situation where the relative distance between a rover and any obstacle boundary is less than rM (safety margin) where $rM<r_{s}$.

The dynamics of the hauler *R* are represented as $\dot{R}=v$. Furthermore, the speed limit of *R* is *S*, i.e., $∥\dot{R}∥\leq S$.

The dynamics of the scouter $R^{s}$ are represented as ${\dot{R}}^{s}=v^{s}$. Note that one does not consider the non-holonomic motion constraint. Furthermore, the speed limit of the scouter $R^{s}$ is $S^{s}$.

While the hauler *R* maneuvers in a dark outer space, it can localize itself utilizing VI-SLAM, which is based on stereo cameras and IMU [6,7,12]. Stereo cameras and IMU are used for localization of the hauler, while it moves. In the situation where the hauler uses Lidar only, its localization error may increase fast while it moves. The authors of [12] also used Lidar, IMU, and stereo cameras for rover localization. Note that among all rovers, only the hauler *R* can locate itself utilizing VI-SLAM. In our paper, both the scouter and the excavator do not have to run localization process, which can be computationally heavy.

It is assumed that the hauler *R* can measure its travel distance using its odometry systems. Whenever *R* travels Δ distance starting from the base station, *R* returns to the base station for initializing the location error to zero. This homing approach was also used in [12]. As *R* is near the base station whose global position is known in advance, *R* measures the base station using Lidar. The Lidar provides the relative position of the base station with respect to *R*. This relative position information provides the global localization for *R*.

For instance, suppose that the base station is at the origin, and that the relative position information of the base station with respect to *R* is $R_{r}$. In this case, the global position of *R* is estimated as $-R_{r}$.

Whenever the hauler *R* travels Δ distance starting from the base station, the hauler *R* returns to the base station for initializing the location error to zero. Here, Δ can be decided by estimating the localization accuracy of *R*. The authors of [12] used the Extended Kalman Filter (EKF) to estimate the global location of a rover, in the situation where the rover uses both IMU and stereo cameras for locating itself. The EKF provides the error covariance matrix P for the location of the hauler *R*. Let $P_{x}$ and $P_{y}$ stand for the error covariance for the global coordinates (*x*,*y*) of the hauler *R* respectively. Then, $u=\sqrt{P_{x}}+\sqrt{P_{y}}$ provides the uncertainty of the position estimate of *R*. Whenever *u* is bigger than a certain threshold, the hauler *R* returns to the base station for initializing the location error to zero. As the hauler *R* returns to the base station, its error covariance matrix is also re-initialized.

For fully covering the bounded obstacle-rich workspace, the hauler *R* forms *g uidance sensors.* Guidance sensors are used to guide the motions of the hauler *R*. As the hauler *R* forms a guidance sensor, the scouter $R^{s}$ fully scans the open space near *R*. Section 4.3 explains a strategy for fully scanning the open space near the hauler *R*.

Let $v_{1}$ and $v_{2}$ indicate two guidance sensors. Let $v_{1}$ and $v_{2}$ indicate the locations of $v_{1}$ and $v_{2}$, respectively. For convenience, let $l(v_{1},v_{2})$ stand for a straight line connecting two guidance sensors $v_{1}$ and $v_{2}$.

Consider a circle with radius rM centered at $v_{1}$. One says that $l(v_{1},v_{2})$ is *obstacle-free*, in the situation where the circle does not cross obstacles while moving along $l(v_{1},v_{2})$. Let $∥l(v_{1},v_{2})∥$ stand for the length of $l(v_{1},v_{2})$.

We define a graph structure, termed *guidance graph I*, as I=(V(I),E(I)). Here, V(I) is the vertex set and E(I) is the edge set. Every vertex in V(I) indicates a formed guidance sensor. In addition, every edge, say $\left\{v_{1},v_{2}\right\}\in E\left(I\right)$, indicates that $l(v_{1},v_{2})$ is obstacle-free and that $∥l\left(v_{1},v_{2}\right)∥<\eta *r_{s}$. Here, η≥1 is a constant. In MATLAB-based simulations, one uses η=3.

Based on the definition of E(I), the hauler *R* evades collision with obstacles while moving along an edge in E(I). Therefore, *I* is an obstacle-free topological map for *R*.

The hauler *R* can find a shortest path between any two vertices by accessing *I*. Increasing η can decrease the path length between any two vertices, as *R* searches for a shortest path using *I*. However, as η increases, the density of *I* increases, since more edges are generated by increasing η. This implies that the computational load increases as η increases.

**Figure 4:**
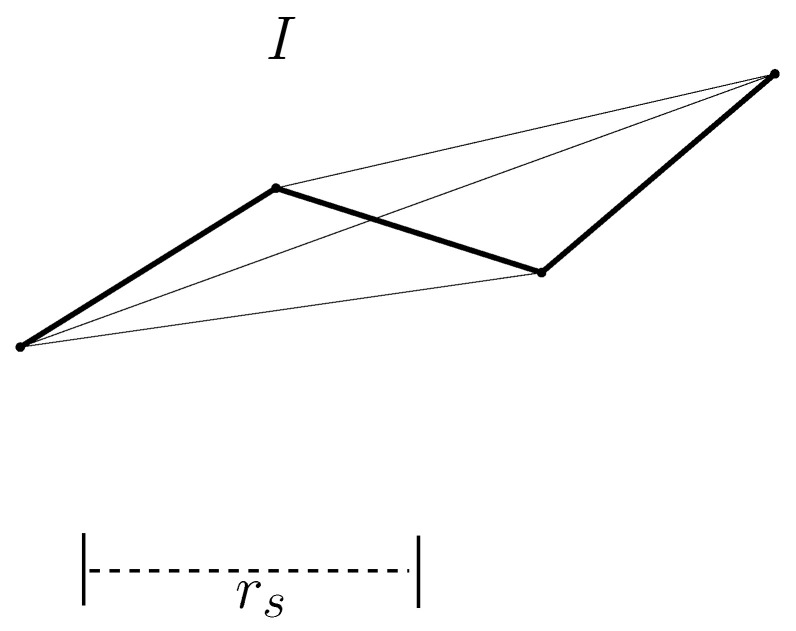
This figure represents *I* as η changes. The length of $r_{s}$ is plotted as a dashed line at the bottom of the figure. If η=1.1, then *I* consists of three edges (three bold edges in the figure). However, if η=3, then *I* consists of six edges.

See Figure 4 as an example. This figure plots *I* as η changes. In Figure 4, the length of $r_{s}$ is plotted as a dashed line at the bottom of the figure. If η=1.1, then *I* consists of three edges (three bold edges in Figure 4). However, if η=3, then *I* consists of six edges.

Whenever the hauler *R* forms a new guidance sensor, *I* is updated to contain the guidance sensor. The weight of $\left\{v_{1},v_{2}\right\}$ indicates $∥l(v_{1},v_{2})∥$. By accessing the weight for every edge in *I*, *R* can find an obstacle-free shortest path from one guidance sensor to another.

A point set $s\in R^{2}$ satisfying that $∥R-s∥=r_{s}$ is called the *footprint*. Recall that $r_{s}$ stands for the sensing range limit of a rover.

An *open footprint arc* of a guidance sensor stands for an arc of a footprint along an open space without obstacles. A *frontier* of a guidance sensor is the subset of an open footprint arc, such that every point along the frontier is outside the footprint of any other guidance sensor. Thus, as every guidance sensor senses no frontier, every guidance sensor fully covers the bounded obstacle-rich workspace.

The footprint is discretized with discrete points, called *footprintPnts*. One generates *D* footprintPnts along the footprint. As *D* increases, one increases the density of footprintPnts along the footprint. In MATLAB-based simulations, one uses D=6. Among all footprintPnts, one searches for a footprintPnt satisfying the following conditions:

- the footprintPnt is on a frontier.
- the straight line connecting *n* and the footprintPnt is an obstacle-free path.

The found footprintPnt is termed the *FrontierVertex* of *n*. Let f(n) stand for the FrontierVertex of *n*. The hauler *R* utilizes a FrontierVertex as a “guidancePoint” for fully scanning the bounded obstacle-rich workspace, without colliding with obstacles.

## 4. Scanning Path Plan Strategy

This article studies how to fully scan an unknown workspace, as fast as possible utilizing the scouter and the hauler. The proposed scanning path planner appears in Algorithm 1. As the hauler senses no guidance sensor inside the footprint, the hauler forms a new guidance sensor. The hauler *R* calculates the shortest path in *I* from its current location to every guidance sensor with a FrontierVertex. Among these guidance sensors with a FrontierVertex, the hauler *R* finds the closest one, say $f_{R}$.

The hauler tracks the shortest path to $f_{R}$. While the hauler *R* approaches $f_{R}$, both the excavator $R^{e}$ and the scouter $R^{s}$ follow *R*. Section 4.5 presents how to make a rover follow the hauler *R*.

One presents the path planner of the hauler *R* for arriving at $f_{R}$. Suppose the hauler *R* plans to encounter guidance sensors $n_{1}\to n_{2}\to ...\to n_{end}$ in this order. Here, $n_{end}$ is the guidance sensor that has $f_{R}$ as its FrontierVertex.

Since this path $n_{1}\to n_{2}\to ...\to n_{end}$ is a subset of *I*, this path is obstacle-free for the hauler *R*. Furthermore, the length of each line segment along this path is shorter than $r_{s}$. After the hauler *R* encounters $n_{j}$ where j≤end−1, *R* approaches $n_{j+1}$. Repeat this maneuvers until the hauler *R* encounters $n_{end}$. After the hauler *R* encounters $n_{end}$, *R* approaches $f_{R}$.

Once the hauler *R* reaches $f_{R}$, it forms another guidance sensor. Since a new guidance sensor is formed, several FrontierVertices inside the sensing range limit of the guidance sensor are removed. The hauler *R* then searches for the nearest FrontierVertex by accessing *I*. The hauler *R* repeats this maneuver, until it can’t detect any other FrontierVertex. Here, *R* accesses *I* for detection of a FrontierVertex.

**Figure 5:**
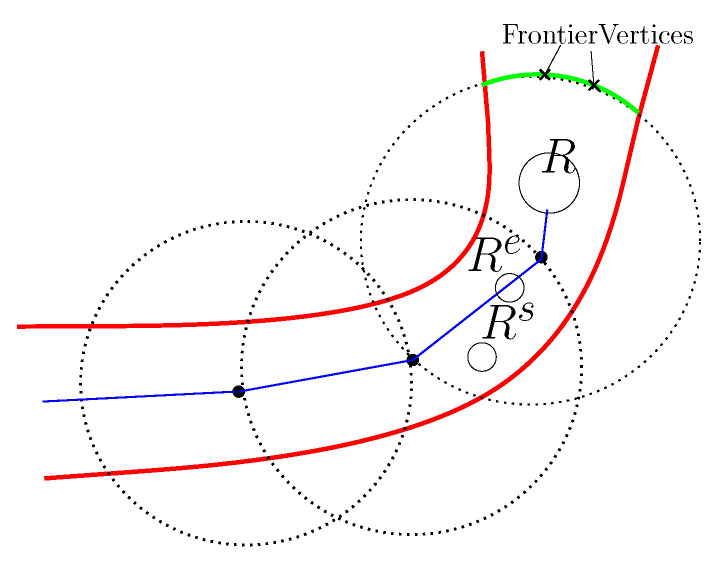
The hauler *R* forming a new guidance sensor. In this figure, *R*, $R^{e}$, and $R^{s}$ are shown as circular robots. The obstacle boundaries are plotted as red curves. The path of the hauler *R* is shown as blue lines. The large dots along the path of the hauler *R* illustrate the guidance sensors formed by the hauler *R*. The footprint of each guidance sensor is represented as a dotted circle. A frontier of the recently formed guidance sensor is shown as a green curve. Two FrontierVertices are shown as two crosses along the frontier.

Figure 5 illustrates the hauler forming a new guidance sensor. The obstacle boundaries are plotted as red curves. The path of the hauler *R* is shown as blue lines. The large dots along the path of the hauler *R* illustrate the guidance sensors formed by the hauler *R*. The footprint of each guidance sensor is plotted as a dotted circle. A frontier of the recently formed guidance sensor is plotted as a green curve. Two FrontierVertices are shown as two crosses along the frontier.

Algorithm 1 states that whenever the hauler *R* travels Δ distance starting from the base station, *R* tracks the shortest path in *I* for reaching the base station. Once the rover is near the base station, it turns on its Lidar to measure the relative position of the base station. In this way, the rover fixes its localization error whenever it homes to the base station. The authors of [12] showed that this homing strategy diminishes the integrated location error to zero.

We have the following theorem regarding the convergence of Algorithm 1.

**Theorem 1.**  *All rovers move under Algorithm 1. In the situation where the hauler R can’t sense any frontier, the complete open space is scanned by footprints.*

**Proof.**  Under the transposition rule, one proves the following statement: If an unexplored open space exists under Algorithm 1, then the hauler *R* senses a frontier associated to the unexplored open space.

Suppose an unexplored open space, say openS, exists, while all rovers move under Algorithm 1. Recall that a frontier of a guidance sensor stands for a subset of an open footprint arc, such that every point along the frontier is outside the footprint of any other guidance sensor. Hence, there exists a guidance sensor, say *n*, which has a frontier along the boundary of this unexplored open space openS. Thus, the hauler *R* senses this frontier associated to openS.    □

**Algorithm 1** Scanning path plan strategy
All rovers are at the base station;
**repeat**
    **if** the hauler *R* senses no guidance sensor inside the footprint **then**         Scan(R);    **end if**    **if** $f_{R}$ is empty **then**         FindFrontierVertex(R);    **end if**    **if** the hauler *R* encounters $f_{R}$ **then**         Scan(R);         FindFrontierVertex(R);    **end if****until** there is no FrontierVertex for every guidance sensor;


### 4.1. Collision Evasion of the Hauler R while Approaching $f_{R}$

In Algorithm 1, the hauler *R* tracks a path for reaching $f_{R}$. Let $\left\{n_{1}\to n_{2}\to \ldots \to n_{end}\right\}$ stand for the order of guidance sensors along the path for reaching $f_{R}$. After meeting $n_{j}$ (j≤end−1), the hauler *R* approaches $n_{j+1}$. One shows that collision evasion is guaranteed, while the hauler *R* approaches $f_{R}$.

Note that $\left\{n_{1}\to n_{2}\to \ldots \to n_{end}\right\}$ is the subset of *I*. According to the definition of E(I), this path is an obstacle-free path. After meeting $n_{end}$, the hauler *R* approaches $f_{R}$. According to the definition of a FrontierVertex, the hauler *R* evades colliding with obstacles while it moves from $n_{end}$ to $f_{R}$.

### 4.2. Controller of the Hauler

In this subsection, one addresses the controller of the hauler. One derives the controller for the hauler *R* in discrete-time systems.

Suppose W is the next guidancePoint that the hauler *R* will encounter along the path to $f_{R}$. Let R(k) stand for the 2D position of the hauler *R* at sample-stamp *k*. Let v(k) stand for the velocity of the hauler *R* at sample-stamp *k*. The dynamics of the hauler *R* are represented as

(1) $$\begin{array}{c} R(k+1)=R(k)+v(k)*T=R(k)+\beta *g(k)*T \end{array}$$

where g(k)=W−R(k). In Equation (1), *T* stands for the sampling interval. In Equation (1), β>0 is a tuning parameter. Equation (1) implies that the hauler *R* approaches W with a speed proportional to ∥g(k)∥.

The speed limit of the hauler *R* is *S*. Therefore, one handles the situation where β∗∥g(k)∥>S in Equation (1). In the situation where β∗∥g(k)∥>S, Equation (1) is replaced by

(2) $$\begin{array}{c} R\left(k+1\right)=R\left(k\right)+v\left(k\right)T=R\left(k\right)+S\frac{g(k)}{∥g(k)∥}T. \end{array}$$

### 4.3. Control Law of the Scouter

Only the scouter has sensors for detecting resources buried in the ground using its local sensors. Thus, only the scouter has the ability to scan the space searching for buried resources.

Whenever the hauler *R* forms a guidance sensor in Algorithm 1, the scouter $R^{s}$ fully scans the open space near *R*. One addresses how the scouter $R^{s}$ fully scans the open space near the hauler *R*.

Recall that every footprint has radius $r_{s}$. Let *ScanCircle* stand for a circle with radius $\frac{r_{s}}{\sqrt{3}}$ centered at the hauler *R*. The area inside this circle stands for the area that will be fully scanned by the scouter $R^{s}$.

**Figure 6:**
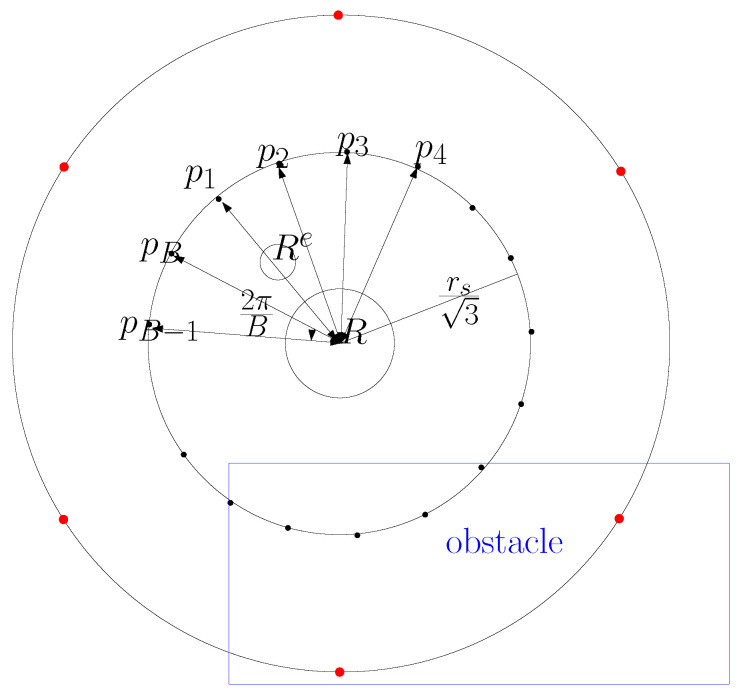
ScanCirclePnts are depicted along the ScanCircle with radius $\frac{r_{s}}{\sqrt{3}}$. In this figure, D=6 footprintPnts are marked with red dots along the footPrint centered at the hauler *R*. There is a rectangular obstacle (blue box).

The ScanCircle is discretized as evenly spaced points, called *ScanCirclePnts*. Suppose there are *B* ScanCirclePnts along the ScanCircle. Here, B>0 is a tuning constant in this research. As *B* increases, one increases the density of ScanCirclePnts along the ScanCircle. See Figure 6 for an illustration. In Figure 6, ScanCirclePnts are depicted along the ScanCircle with radius $\frac{r_{s}}{\sqrt{3}}$. In this figure, D=6 footprintPnts are depicted with red dots along the footPrint centered at the hauler *R*. There is a rectangular obstacle (blue box) in Figure 6.

Let $p_{1}$ stand for the ScanCirclePnt, where $l(R,R^{s})$ crosses the footprint. One then enumerates all ScanCirclePnts starting from $p_{1}$, as plotted in Figure 6.

The scouter $R^{s}$ maneuvers backwards and forwards between $p_{i}$ (i∈{1,2,…,B}) and the hauler *R* to fully scan all points on $l(p_{i},R)$. For fully scanning the open space near the hauler *R*, the scouter $R^{s}$ visits the ScanCirclePnts and R in the following order: $p_{1}\to R\to p_{2}\to R\ldots \to p_{B}\to R$.

Let $v^{s}\left(k\right)$ stand for the velocity of the scouter $R^{s}$ at sample-stamp *k*. The dynamics of the scouter $R^{s}$ while approaching $p_{i}$ are represented as

(3) $$\begin{array}{c} R^{s}\left(k+1\right)=R^{s}\left(k\right)+v^{s}\left(k\right)*T, \end{array}$$

where

(4) $$\begin{array}{c} v^{s}\left(k\right)=S^{s}*\frac{p_{i}-R}{∥p_{i}-R∥}. \end{array}$$

Equation (4) implies that the scouter $R^{s}$ moves in the direction of $p_{i}-R$ with its speed limit $S^{s}$. The scouter $R^{s}$ keeps moving in the direction of $p_{i}-R$ until $∥R^{s}-R∥=r_{s}$ is satisfied. Once $∥R^{s}-R∥=r_{s}$ is satisfied, the scouter $R^{s}$ gets back to the hauler *R*.

While the scouter $R^{s}$ approaches $p_{i}$, the scouter $R^{s}$ may be too close to an obstacle, such as a rectangular obstacle in Figure 6. Whenever the scouter $R^{s}$ gets too close to an obstacle, it gets back to the hauler *R*.

Once the scouter $R^{s}$ encounters the hauler *R*, the scouter $R^{s}$ begins approaching $p_{i+1}$. This maneuver repeats, while *i* increases in the following order: {1→2→…→B}. Once *i* becomes *B*, the open space near the hauler *R* has been scanned completely.

### 4.4. Control Law of the Excavator

Suppose that the scouter $R^{s}$ detects buried resources while maneuvering backwards and forwards between $p_{j}$ (j∈{1,2,…,B}) and *L* in Algorithm 2. Whenever the scouter $R^{s}$ detects a place where resources are buried, then the hauler *R* comes to the place, while the excavator $R^{e}$ follows the hauler *R*.
**Algorithm 2**  
Scan(R)
The hauler *R* forms a guidance sensor at its location, say *L*;By maneuvering backwards and forwards from *L*, the scouter $R^{s}$ fully scans the open space near the hauler *R* (see Section 4.3 for controls of the scouter);**if** The scouter $R^{s}$ senses a place where resources are buried **then**   The hauler *R* arrives at the place, while the excavator $R^{e}$ follows *R*;   The excavator $R^{e}$ excavates the resources, and drops the resources into the bin of *R* (see Section 4.4 for controls of the excavator);   The hauler *R* gets back to *L*, while the excavator $R^{e}$ follows the hauler *R* (see Section 4.5 for controls of following maneuvers);**end if**

Thereafter, the excavator $R^{e}$ excavates the found resources under digging controls in [12,27,28,29]. Then, the excavator $R^{e}$ drops the found resources into the bin of hauler *R* under excavator controls in [12,27,28,29]. Note that developing digging controls is not within the scope of this paper.

Then, the hauler *R* gets back to *L*, while the excavator $R^{e}$ follows the hauler *R*. For making a rover follow the hauler *R*, one can use deep neural networks for hauler detection [12,30], as well as robot tracking controls in [31,32,33,34,35]. Section 4.5 presents how to make a rover follow the hauler *R*.

In this way, the excavator can dig all resources surrounding *L* in Algorithm 2. Suppose that natural resources are concentrated in a specific region surrounding *L*. In this case, the hauler can become full, while the excavator drops the resources into the bin of the hauler.

Whenever the hauler is full of resources, it transfers the resources to the base station. While the hauler maneuvers for transferring the resources to the base station, both the scouter and the excavator wait at their positions. After the resource transfer is done, the hauler comes back to the waiting positions of both the scouter and the excavator. Thereafter, the rover team restarts scanning of the space.

### 4.5. Following Maneuvers of a Rover

For making a rover follow the hauler, we need to train the rover’s stereo camera using deep neural networks [12,30]. Object detection strategy using neural networks was also used in [12]. In [12], the networks were trained to identify various classes, such as processing plant, rocks, visible volatiles above or partially above the surface, craters, and the other rovers. Once the networks are trained, the rover can detect the hauler’s image in the camera view. Also, the rover can detect the relative distance to the hauler using the stereo camera image.

Suppose that the hauler is detected in the rover’s camera image. For making a rover follow the hauler, one can use the tracking controls in [31,32,33,34,35]. References [31,32,33,34,35] designed tracking control laws to let a rover follow another object based on local sensing measurements.

## 5. Matlab-Based Simulations

To the best of our knowledge, our article is novel in addressing the scanning path plan, so that the rover team fully scans a given workspace while fixing the location error occasionally. One demonstrates the efficacy of Algorithm 1 through MATLAB-based simulations.

The simulation parameters are set as follows. The sensing range $r_{s}$ is set as 3 distance units. The sampling time interval *T* is set as 0.1 s.

One sets rM=0.5 distance unit. The speed limit of a rover is 10 distance units per second. One sets *D*, the number of footprintPnts on a footprint, as 6. Furthermore, one sets *B*, the number of ScanCirclePnts on a footprint, as 14.

### 5.1. Scenario 1

In Scenario 1, we generate obstacle environments, inspired by Figure 2. Rocks in Figure 2 are presented as obstacles in Scenario 1. Reference [12] didn’t address a strategy for planning the path of rover team, so that the given workspace is fully scanned without detection holes.

#### 5.1.1. The Ideal Situation where There Is no Localization Error

In Scenario 1, we generate obstacle environments, inspired by Figure 2. Considering Scenario 1, one first simulates the ideal situation where there is no localization error. One sets Δ=∞ in Algorithm 3.

**Figure 7:**
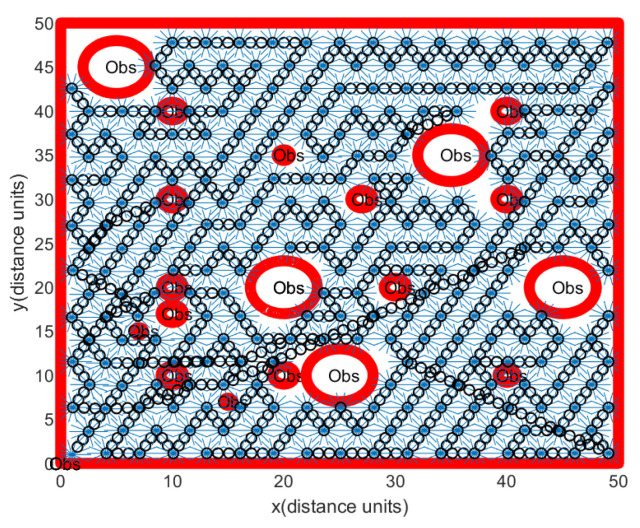
Scenario 1. In this scenario, we generate obstacle environments, inspired by Figure 2. There is no localization error. One sets Δ=∞ in Algorithm 3. Obstacle boundaries are shown with thick red curves. The red rectangle illustrates the workspace boundary. The path of the hauler *R* is depicted as a black circle. Backwards and forwards maneuvering of the scouter $R^{s}$ (blue lines) is used to scan a ScanCircle entirely.

Regarding Scenario 1, Figure 7 illustrates a rover’s path constructed through Algorithm 1. Obstacle boundaries are shown with thick red curves. In Figure 7, Obs illustrates the obstacles. The red rectangle represents the workspace boundary. At sample-stamp 0, the position of the hauler *R* is (1,1), which is the position of the base station. The path of the hauler *R* is marked as a black circle. Backwards and forwards maneuvering of the scouter $R^{s}$ (blue lines in Figure 7) is used to scan a ScanCircle entirely. Figure 7 shows that footprints of all guidance sensors cover the complete open space.

**Figure 8:**
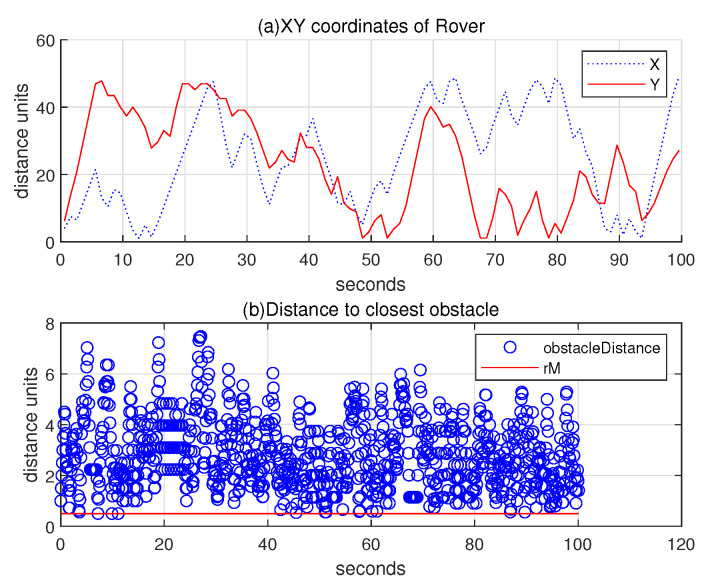
Scenario 1. Regarding the scenario in Figure 7, the upper subplot represents the 2D coordinates of the hauler with respect to time, while the lower subplot illustrates the relative distance between the hauler *R* and its closest obstacle boundary with respect to time. In the scanning process, the hauler’s distance to the nearest obstacle boundary always exceeds rM=0.5 distance units.

Regarding the scenario in Figure 7, the upper subplot of Figure 8 illustrates the 2D coordinates of the hauler with respect to time, while the lower subplot of Figure 8 illustrates the relative distance between the hauler *R* and its nearest obstacle boundary with respect to time. In the scanning process, the hauler’s distance to the nearest obstacle boundary always exceeds rM=0.5 distance units.
**Algorithm 3**  
FindFrontierVertex(R)
The hauler *R* searches for the nearest FrontierVertex by accessing *I*;The nearest FrontierVertex is set as $f_{R}$;The hauler *R* tracks the shortest path in *I* for reaching $f_{R}$;The excavator $R^{e}$ and the scouter $R^{s}$ follow the hauler *R* (see Section 4.5 for controls of following maneuvers);**if** the hauler *R* has traveled Δ distance starting from the base station **then**   The hauler *R* tracks the shortest path in *I* for reaching the base station, while the scouter $R^{s}$ and the excavator $R^{e}$ follow *R* (see Section 4.5 for controls of following maneuvers);   Set $f_{R}=\emptyset$;**end if**

#### 5.1.2. The Situation where There Is Localization Error

In Scenario 1, we generate obstacle environments, inspired by Figure 2. Considering Scenario 1, one next considers the situation where there is localization error. Due to the localization error, the dynamics of the hauler *R* may not be described using (1). Regarding the environmental perturbation (e.g., wheel slippage), the true dynamics of the hauler *R* are represented as

(5) $$\begin{array}{c} R^{p}\left(k+1\right)=R^{p}\left(k\right)+v\left(k\right)*T+n \end{array}$$

where n stands for the process noise due to the environmental perturbation. Here, $R^{p}$ is used instead of R, in order to emphasize that $R^{p}$ is the perturbed rover position. In simulations, n is a Gaussian distribution with mean 0 and variance $\sqrt{0.00005}$. Since the hauler *R* can’t access the process noise term n, the location estimation of the hauler *R* is updated using (1) instead of (5).

At sample-stamp 0, $R^{p}\left(0\right)=R\left(0\right)=\left(1,1\right)$ which is the position of the base station. Suppose that the hauler *R* returns to the base station at sample-stamp *k*. Then, one sets $R^{p}\left(k\right)=R\left(k\right)=\left(1,1\right)$. This implies that the hauler’s location estimation error is reset to zero at sample-stamp *k*.

**Figure 9:**
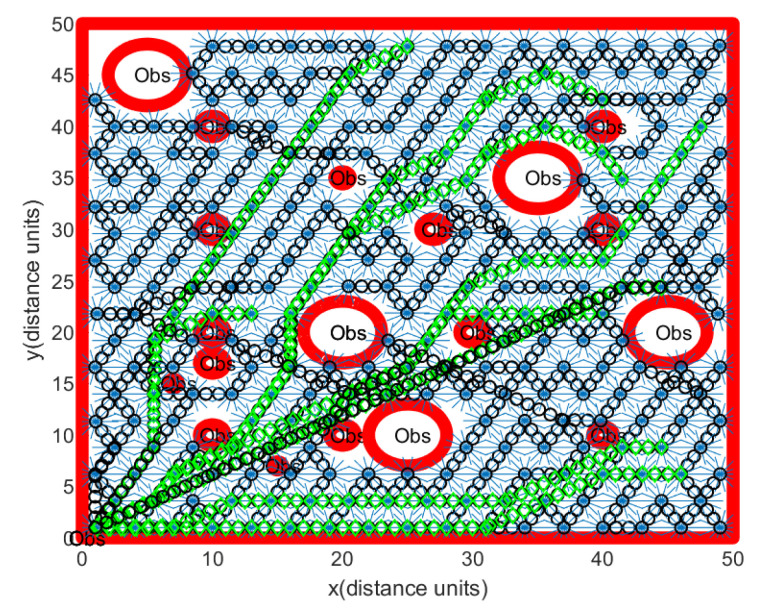
Scenario 1 with localization error. In Scenario 1, we generate obstacle environments, inspired by Figure 2. One sets Δ=100 in Algorithm 3. The path of the hauler *R* is marked as a black circle. The hauler *R* occasionally homes to the base station by applying Δ=100 in Algorithm 3. The homing maneuver of the hauler *R* is depicted with green diamonds. Backwards and forwards maneuvering of the scouter $R^{s}$ (blue lines) is used to scan a ScanCircle entirely.

One sets Δ=100 in Algorithm 3. Regarding Scenario 1 with localization error, Figure 9 illustrates a rover’s path constructed through Algorithm 1. At sample-stamp 0, the position of the hauler *R* is (1,1) which presents the position of the base station. The path of the hauler *R* is marked as a black circle. The hauler *R* occasionally homes to the base station by applying Δ=100 in Algorithm 3. The homing maneuver of the hauler *R* is marked with green diamonds in Figure 9. Backwards and forwards maneuvering of the scouter $R^{s}$ (blue lines in Figure 9) is used to scan a ScanCircle entirely. Figure 9 shows that footprints of all guidance sensors cover the complete open space.

**Figure 10:**
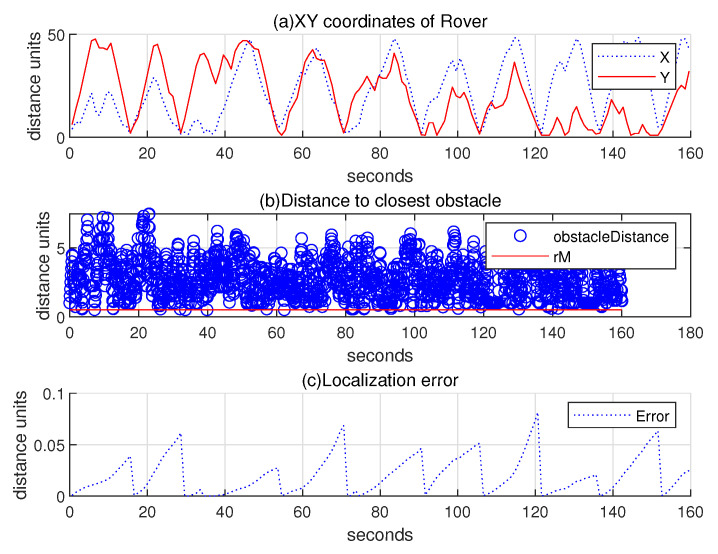
Scenario 1 with localization error. Regarding the scenario in Figure 9, (**a**) subplot plots the 2D coordinates of the hauler with respect to time, while (**b**) subplot illustrates the relative distance between the hauler *R* and its closest obstacle boundary with respect to time. In the scanning process, the hauler’s distance to the nearest obstacle boundary always exceeds rM=0.5 distance units. Compared to Figure 8, the scanning time increases considerably, since the hauler *R* needs to home to the base station occasionally. (**c**) subplot presents the localization error $∥R\left(k\right)-R^{p}\left(k\right)∥$ as *k* varies.

Regarding the scenario in Figure 9, (a) subplot of Figure 10 illustrates the 2D coordinates of the hauler with respect to time, while (b) subplot illustrates the relative distance between the hauler *R* and its nearest obstacle boundary with respect to time. In the scanning process, the hauler’s distance to the nearest obstacle boundary always exceeds rM=0.5 distance units. Compared to Figure 8, the scanning time increases considerably, since the hauler *R* needs to home to the base station occasionally. (c) subplot presents the localization error $∥R\left(k\right)-R^{p}\left(k\right)∥$ as *k* varies. See that the location error is reset to zero, whenever the hauler *R* returns to the base station.

#### 5.1.3. Comparison with a Random Maneuver Strategy

As far as we know, only [12] addressed the exploration of three rovers (the scouter, the hauler, and the excavator), as presented in our study. However, [12] didn’t present a strategy for fully scanning the bounded obstacle-rich workspace, as presented in our study.

**Figure 11:**
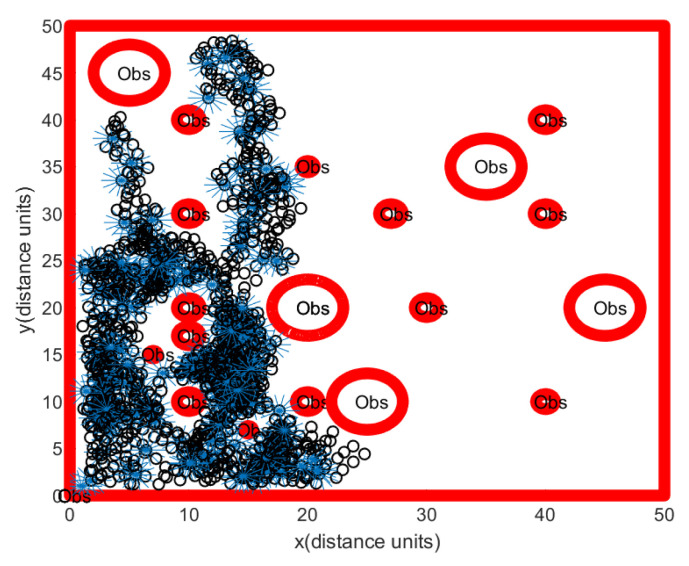
Scenario 1 with localization error under the random maneuver strategy. In Scenario 1, we generate obstacle environments, inspired by Figure 2. The path of the hauler *R* is marked as a black circle. Backwards and forwards maneuvering of the scouter $R^{s}$ (blue lines) is used to scan a ScanCircle entirely. See that the bounded obstacle-rich workspace can’t be fully scanned by a random maneuver. The simulation ends after 150 s pass.

For comparison, one simulates a group of rovers, so that they randomly maneuver in the bounded obstacle-rich workspace. References [23,24,25] showed that random maneuvers can be used to find resources in the workspace. Suppose that rovers move as a group and that they move randomly in the workspace, while avoiding collision with obstacles. Whenever the distance between a rover and an obstacle is less than $2*S^{s}*T$, the rover moves away from the obstacle with speed $S^{s}$. Whenever the hauler *R* moves for $S^{s}*T$ distance, the scouter $R^{s}$ fully scans the open space near *R* using the clearing strategy in Section 4.3. Regarding Scenario 1 with localization error, Figure 11 illustrates a rover’s path constructed through this random maneuver strategy. See that the bounded obstacle-rich workspace can’t be fully scanned by a random maneuver. The simulation ends after 150 s pass.

Figure 11 shows that rovers under the random maneuver strategy can’t explore the bounded obstacle-rich workspace, since random maneuver does not assure that they cover the bounded obstacle-rich workspace. We argue that this random maneuver strategy can’t make the rovers move through a narrow passage. We show that the proposed scanning strategy in Algorithm 3 outperforms this random maneuver strategy.

**Figure 12:**
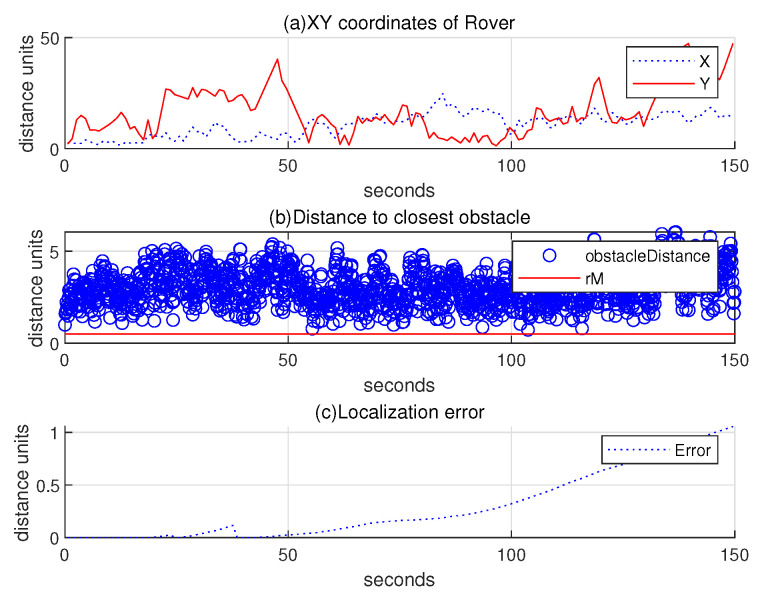
Scenario 1 with localization error under the random maneuver strategy. Regarding the scenario in Figure 11, (**a**) subplot plots the 2D coordinates of the hauler with respect to time, while (**b**) subplot illustrates the relative distance between the hauler *R* and its closest obstacle boundary with respect to time. In the scanning process, the hauler’s distance to the nearest obstacle boundary always exceeds rM=0.5 distance units. (**c**) subplot presents the localization error $∥R\left(k\right)-R^{p}\left(k\right)∥$ as *k* varies.

Regarding the scenario in Figure 11, (a) subplot of Figure 12 illustrates the 2D coordinates of the hauler with respect to time, while (b) subplot illustrates the relative distance between the hauler *R* and its nearest obstacle boundary with respect to time. In the scanning process, the hauler’s distance to the nearest obstacle boundary always exceeds rM=0.5 distance units. (c) subplot presents the localization error $∥R\left(k\right)-R^{p}\left(k\right)∥$ as *k* varies. While the rovers randomly maneuver, they can get close to the base station. In this case, their location error is reset by chance.

### 5.2. Scenario 2

In Scenario 2, we consider environments with large obstacles. Regarding Scenario 2 with localization error, the dynamics of the hauler *R* are represented using (5). At sample-stamp 0, $R^{p}\left(0\right)=R\left(0\right)=\left(1,1\right)$ which is the position of the base station. Suppose that the hauler *R* returns to the base station at sample-stamp *k*. Then, one sets $R^{p}\left(k\right)=R\left(k\right)=\left(1,1\right)$.

**Figure 13:**
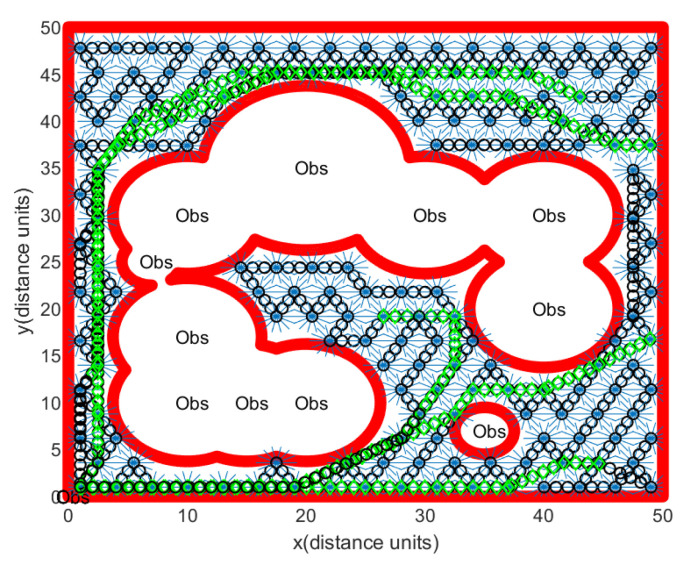
Scenario 2 with localization error. One sets Δ=100 in Algorithm 3. The path of the hauler *R* is depicted as a black circle. The hauler *R* occasionally homes to the base station by applying Δ=100 in Algorithm 3. The homing maneuver of the hauler *R* is marked with green diamonds. Backwards and forwards maneuvering of the scouter $R^{s}$ (blue lines) is used to scan a ScanCircle entirely.

One sets Δ=100 in Algorithm 3. Regarding Scenario 2 with localization error, Figure 13 illustrates a rover’s path constructed through Algorithm 1. At sample-stamp 0, the position of the hauler *R* is (1,1) which presents the position of the base station. The path of the hauler *R* is marked as a black circle. The hauler *R* occasionally homes to the base station. The homing maneuver of the hauler *R* is marked with green diamonds in Figure 13. Backwards and forwards maneuvering of the scouter $R^{s}$ (blue lines in Figure 13) is used to scan a ScanCircle entirely.

**Figure 14:**
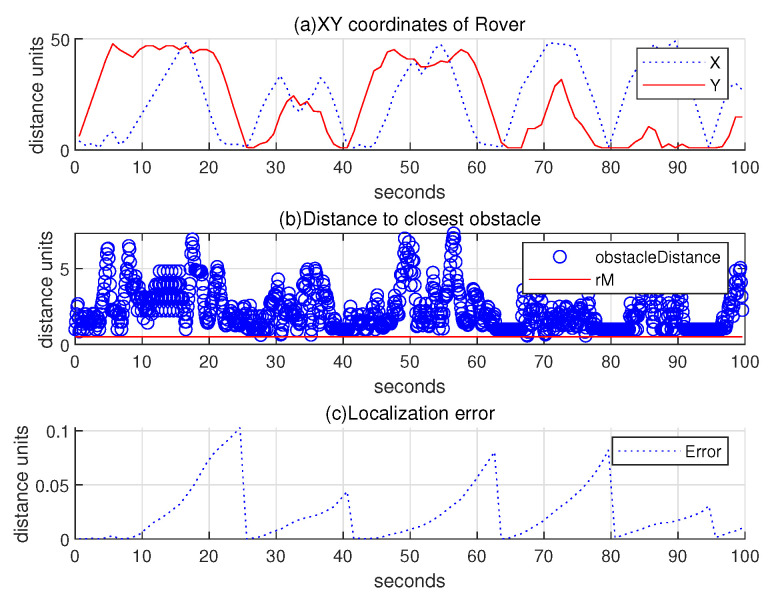
Scenario 2 with localization error. Regarding the scenario in Figure 13, (**a**) subplot illustrates the 2D coordinates of the hauler with respect to time, while (**b**) subplot illustrates the relative distance between the hauler *R* and its closest obstacle boundary with respect to time. (**c**) subplot presents the localization error $∥R\left(k\right)-R^{p}\left(k\right)∥$ as *k* varies.

Regarding the scenario in Figure 13, (a) subplot of Figure 14 represents the 2D coordinates of the hauler with respect to time, while (b) subplot illustrates the relative distance between the hauler *R* and its nearest obstacle boundary with respect to time. In the scanning process, the hauler’s distance to the nearest obstacle boundary always exceeds rM=0.5 distance units. (c) subplot presents the localization error $∥R\left(k\right)-R^{p}\left(k\right)∥$ as *k* varies. See that the location error is reset to zero, whenever the hauler *R* returns to the base station.

#### Comparison with a Random Maneuver Strategy

For comparison, one simulates a group of rovers, so that they randomly maneuver in the bounded obstacle-rich workspace. Refer to Section 5.1.3 for the random maneuver strategy.

**Figure 15:**
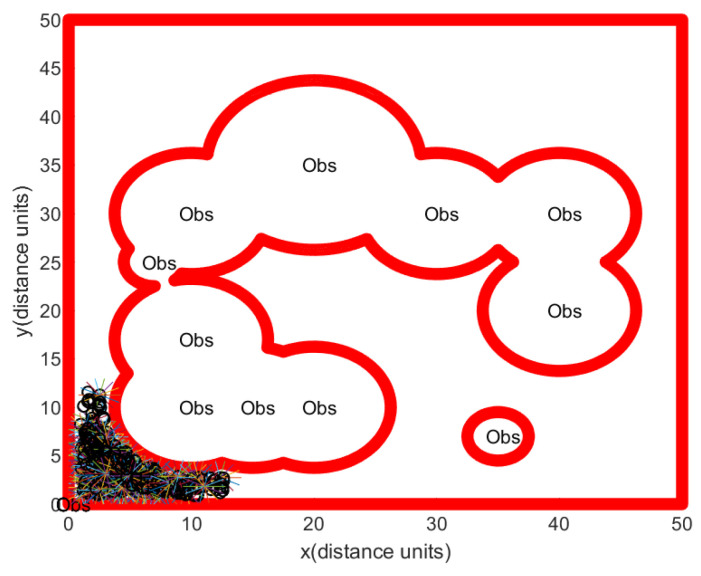
Scenario 2 with localization error under the random maneuver strategy. The path of the hauler *R* is marked as a black circle. Backwards and forwards maneuvering of the scouter $R^{s}$ (blue lines) is used to scan a ScanCircle entirely. See that the bounded obstacle-rich workspace can’t be fully scanned by a random maneuver.

Regarding Scenario 2 with localization error, Figure 15 illustrates a rover’s path constructed through this random maneuver strategy. See that the bounded obstacle-rich workspace can’t be fully scanned by a random maneuver. The simulation ends after 150 s pass.

Figure 15 shows that rovers under the random maneuver strategy can’t explore the bounded obstacle-rich workspace, since random maneuver does not assure that they cover the bounded obstacle-rich workspace. We show that the proposed scanning strategy in Algorithm 3 outperforms this random maneuver strategy.

**Figure 16:**
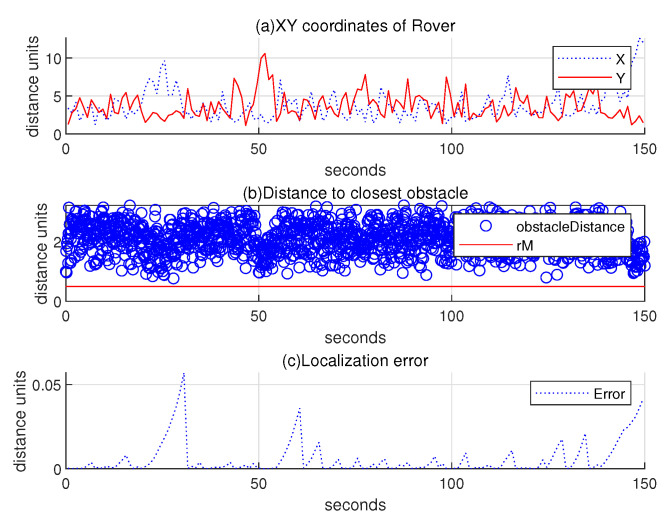
Scenario 2 with localization error under the random maneuver strategy. Regarding the scenario in Figure 15, (**a**) subplot plots the 2D coordinates of the hauler with respect to time, while (**b**) subplot illustrates the relative distance between the hauler *R* and its closest obstacle boundary with respect to time. (**c**) subplot presents the localization error $∥R\left(k\right)-R^{p}\left(k\right)∥$ as *k* varies.

Regarding the scenario in Figure 15, (a) subplot of Figure 16 illustrates the 2D coordinates of the hauler with respect to time, while (b) subplot illustrates the relative distance between the hauler *R* and its nearest obstacle boundary with respect to time. In the scanning process, the hauler’s distance to the nearest obstacle boundary always exceeds rM=0.5 distance units. (c) subplot presents the localization error $∥R\left(k\right)-R^{p}\left(k\right)∥$ as *k* varies. While the rovers randomly maneuver, they can get close to the base station. In this case, their location error is reset by chance.

## 6. Conclusions

This article addresses the scanning path plan strategy of a rover team (the scouter, the hauler, and the excavator) exploring unknown dark outer space environments. The rover team is deployed from a symmetric base station, and the team’s goal is to scan a bounded obstacle-rich workspace entirely. For location estimate fix, the rover occasionally homes to the base station, whose shape and global position are known in advance. Once a rover is near the station, it utilizes its Lidar to measure the relative position of the base station. In this manner, the rover fixes its localization error whenever it homes to the base station.

This article makes the rover team fully scan the given workspace without detection holes, such that a rover’s location error is bounded by letting the rover home to the base station occasionally. To the best of our knowledge, this article is novel in addressing the scanning path plan strategy, so that a rover team fully scans a bounded outer space workspace without detection holes, while fixing the location error occasionally. The efficacy of the proposed scanning and location approach is demonstrated utilizing MATLAB-based simulations. In the future, we will do experiments using real ground robots, in order to verify the proposed scanning and location approach rigorously.

## Data Availability

Data are available from the authors upon reasonable request.
